# Quantifying Annual Nitrogen Loss to Groundwater Via Edge‐of‐Field Monitoring: Method and Application

**DOI:** 10.1111/gwat.13217

**Published:** 2022-06-23

**Authors:** Michael Cardiff, Laura Schachter, Jake Krause, Madeline Gotkowitz, Brian Austin

**Affiliations:** ^1^ Department of Geosciences University of Wisconsin‐Madison Madison WI USA; ^2^ Water Science Center US Geological Survey Wisconsin Middleton WI USA; ^3^ SCS Engineers Madison WI USA; ^4^ Montana Bureau of Mines and Geology Butte MT USA; ^5^ Wisconsin Department of Natural Resources Madison WI USA

## Abstract

Increased nitrate concentrations in groundwater and surface waters represent one of the most widespread and acute impacts of modern agriculture on the environment. However, there is often a fundamental gap in understanding how individual agricultural fields and practices contribute to this broad‐scale issue. To practically address nutrient dynamics at individual agricultural sites, methods for assessing nitrogen loss to groundwater that are minimally invasive and thus can encourage farmer “buy in” are necessary. We present an approach that uses edge‐of‐field monitoring at multilevel samplers along with a once‐per‐year tracer application (bromide) to calculate nitrogen loss on an annual basis. Using appropriate spatio‐temporal integrals of measured concentrations, a net loss of nitrogen to groundwater (per field area) can be calculated. This approach directly measures impacts of nitrogen leaching below the water table, while avoiding permanent in‐field installations that can interfere with farm operations. We present an application of this technique to assess nitrogen loss to groundwater over 5 years for a commercial agricultural field in Sauk County, WI. Results from Field 19 indicate that nitrogen losses are similar to (or slightly below) previously reported values for corn and potato crops. In all years, however, we estimate that more than 25% (>60 kg/ha) of nitrogen applied leached as nitrate to groundwater. Use of this mass flux estimation method was most reliable when: (1) tracer is injected directly at the water table, limiting “smearing” within the vadose zone; and (2) nitrate concentrations from laboratory analysis were obtained, rather than using ion‐selective electrodes or nitrate test strips.

## Abstract 2

*Article Impact Statement*: We demonstrate a minimally invasive monitoring approach that quantifies nitrogen loss to groundwater from an agricultural field over five growing seasons.

## Introduction

Across much of the Midwestern United States, nitrogen fertilizers are applied to agricultural fields in order to increase crop yields. Many of these fields are atop sandy soils and sand or sand‐and‐gravel aquifers, meaning the fields are extremely well drained. These conditions allow both nutrients and water to be lost to the aquifer below, especially during large precipitation events. The nitrogen in fertilizers is often leached in the ionic form of nitrate (NO_3_^−^), which is known to be harmful to infants in high concentrations. Because of these risks, the EPA‐designated maximum contaminant level (MCL) is 10 mg/L nitrate as nitrogen (NO_3_‐N) for drinking water. Additionally, reviews of current human health studies indicate evidence for relationships between drinking water nitrate and colorectal cancer, thyroid disease, and neural tube defects (e.g., see a recent summary in Ward et al. 2018).

The high loading of nitrogen fertilizers in the Midwest, and the relationship between agriculturally active land and increased shallow nitrate concentrations is well‐documented (e.g., Nolan and Stoner 2000). In Wisconsin, in particular, a campaign offering testing of private wells across the state has shown several townships where NO_3_‐N concentrations in groundwater are consistently at or above the MCL (Masarik 2021). Similarly, several communities within Wisconsin are experiencing increasing trends in nitrate concentrations at municipal wells, which will require regulation if the EPA threshold is reached.

The degree to which agricultural practices contribute to nitrate in groundwater depends on a variety of factors including vadose zone and soil properties (including geochemistry), depth to groundwater, hydrostratigraphy, climate, and current and historical agricultural practices. In general, the contribution of individual farms to groundwater nitrate concentrations is poorly known, and the expected impact of changing agricultural practices in reducing nitrate losses to groundwater—or of changing land use on individual parcels—is poorly constrained. Additionally, the storage of “legacy” nitrate in the vadose zone and groundwater complicates relationships between changes to agricultural management practices and impacts to aquatic environments, e.g., the Gulf dead zone (Ascott et al. 2021), meaning that understanding timescales of nitrate transport into and out of groundwater storage volumes is also important for informing management expectations. Many farmers have expressed desires to be “good stewards” for the land and water resources that support their livelihoods and communities. Helping farmers to meet these goals, however, will require methods and instrumentation that can assess individual farms' contribution on an annual basis and assess or guide changes in management, and similarly methods that can estimate the rate of nitrate movement away from agricultural fields toward receptors.

To address this need, over the past several decades researchers have applied different approaches to quantifying annual groundwater nitrogen loading. The most direct methods for loading assessment measure fluxes of nitrogen in soil water immediately before it reaches the water table, or measure nitrogen concentrations in groundwater. Indirect methods—i.e., mass balances—can also be used which measure other components of nitrogen additions and losses and derive nitrogen loss to groundwater as a residual. Here we review approaches described in the literature for quantifying nitrogen (N) loading from individual agricultural fields. While studies of groundwater nitrogen content have been performed in several locations around the world, we focus primarily on studies in North America with similar cropping and soil‐types to those investigated in the current study, in which annually variable N loading estimates to groundwater (kg N/ha) were published.

One approach to estimating N loss to groundwater is via a “mass balance” approach. In this approach, researchers assess N inputs (via fertilizer, atmospheric deposition, and irrigation water) and monitor output N in crop biomass, with the difference representing net N that is transferred to the environment (either soil/root zone, or groundwater). Saffigna et al. (1977) represents an early application of such a mass balance approach at a potato field in Wisconsin. By performing chemical analysis on oven‐dried crop samples, the researchers were able to determine the amount of nitrogen in the biomass of the plant and tuber portions of potatoes. With knowledge of the rates of fertilizer applications to the crops, the researchers were then able to infer total nitrogen loss to the environment, which represents an upper bound on losses to groundwater. If changes to soil zone storage of N is measured annually as well, the net contribution to groundwater can be further refined. Nitrogen mass balance approaches have been applied recently to several European sites in order to estimate nitrogen use efficiency, and these computations showed strong correlations between estimated surplus nitrogen and measured groundwater nitrate concentrations (Dalgaard et al. 2012).

To more directly assess nitrogen content in recharged water, measurements of water chemistry in the unsaturated zone can be performed with porous ceramic tension samplers. Using these samplers, researchers may evaluate fluid chemistry during times when downward flow to the water table is expected (e.g., following storm events). These concentrations are then combined with estimates of downward water volume fluxes to the water table. As one example, Sexton et al. (1996) collected soil water with porous ceramic cups mounted at the end of 60 cm long PVC tubing via vacuum, usually 1–2 days after precipitation or irrigation. To estimate nitrogen loading to groundwater, concentration values were multiplied by modeled drainage fluxes and summed throughout a monitoring year. Sexton et al. used a simple recharge mass balance method that monitored water inputs (precipitation P and irrigation I), estimated evapotranspiration (ET), estimated changes to soil moisture storage (ΔS) via tensiometers, and calculated drainage (recharge, R) as a residual term R=P+I−ET−ΔS. Several other studies have utilized a similar monitoring approach, with some variability in the method used for calculating water drainage to the water table (e.g., Andraski et al. 2000—Wisconsin; Svoboda et al. 2013—Germany; Struffert et al. 2016—Minnesota; De Notaris et al. 2018—Denmark; Hussain et al. 2019—Michigan; Manevski et al. 2015—Denmark; Tully et al. 2012—Costa Rica; Syswerda et al. 2012—Michigan).

Water leaching below the root zones of plants may be captured by either large pan lysimeters or tile drainage systems. Since these tools can capture both the volume of water and the nitrogen content of water exiting the root zone, they provide a valuable estimate of total N mass leaching to groundwater from a given field area. Early examples of monitoring via tile drain and large lysimeters include, respectively, Gast et al. (1978) and Saffigna et al. (1977), and a more recent example from Denmark is Ernstsen et al. (2015). Large lysimeter installations in particular have been employed in several agricultural research fields studies (Rasse et al. 1999; Basso and Ritchie 2005), though their use on commercial farms is limited. An exception is Martin et al. (1994), who employed similar lysimeters at a commercial grower's field, installed at roughly 2 m depth and with a roughly 2 m^2^ monitoring footprint.

While large pan lysimeters represent perhaps the most direct method of measuring nitrate mass loading below the root zone, several experimental issues can degrade the reliability of obtained loading estimates. For one, installation of lysimeters requires the removal of a large quantity of soil and replacement of the soil column (preferably in an “undisturbed” condition) above the apparatus. Excess compaction of soils during this process may lead to under‐estimates of typical field drainage; similarly, pathways for vadose zone flow may be created by soil disturbances that over‐represent drainage. Even if soil is removed and replaced appropriately, the vadose zone conditions created in the vicinity of the lysimeter can strongly affect the net drainage to the instrument. Instead of commonly applied zero‐tension or fixed‐tension lysimeters, these issues can be ameliorated by automated “equilibrium tension lysimeters” (Brye et al. 1999), which have been applied to nitrogen leaching monitoring recently (Masarik et al. 2014).

The porous cup and pan lysimeter methods of assessing N impact to groundwater rely on the assumption that minimal changes to nitrate concentrations will occur once water is below the water table. In areas with favorable accessibility (e.g., shallow depth to groundwater), however, groundwater nitrate (NO_3_‐N) concentrations may be directly observed beneath fields. Stites and Kraft (2001), and later Kraft and Stites (2003), provide an example of a “water year” technique in which multilevel samplers (MLSs) placed on the field allowed observation of concentrations at 19 different elevations within groundwater. These multilevel samplers were strategically placed more than 1 year's groundwater travel distance from any upgradient field edge, so the concentrations sampled would represent water recharged on the field of interest during the current water year. Nitrate as N concentrations $C_{NO_{3}-N}$ can then be converted to a consistent units set (e.g., $\mathrm{kg}/m^{3}$) and integrated over appropriate elevations to compute annual groundwater nitrogen loading, working under the assumption that groundwater flow is primarily horizontal and new recharge arrives in a “layer cake” fashion. Under these assumptions, Kraft and Stites showed that total nitrate leached from an area over a growing year can be computed as:

(1) $$\frac{M}{A}=\theta \int_{z_{1}}^{z_{2}}C_{NO_{3}-N}\mathrm{dz}$$

where θ represents an estimated porosity, and $\frac{M}{A}$ is the mass of nitrate‐N loading per area of the field. The elevations over which integration take place ($z_{1}$ and $z_{2}$) represent the elevations that bound 1 year of recharged water, and were determined in these prior studies using a natural tracer of chloride from a large annual dose of KCl fertilizer that moved to the water table each year.

Instrumentation installed on agricultural fields can complicate (and/or be unintentionally damaged by) agricultural operations. For this reason, monitoring strategies that require minimal field disturbance or on‐field equipment are desirable. Most recently, Malekani et al. (2018)—following a study by Kuipers et al. (2014)—discussed the use of multilevel edge‐of‐field sampling along with tracer testing in order to isolate N leaching occurring from a particular field. This approach has an advantage in that, unlike the “water year” approach pioneered by Stites and Kraft (2001), no instrumentation is installed on the agricultural field. Conceptually, the setup of Malekani et al. (2018) allows computations similar to those used by “flux fences” applied in contaminated site monitoring (e.g., Cai et al. 2012).

A summary of those studies that measured leaching beneath commercial agricultural fields and/or applied conventional farming recommendations to experimental agricultural fields is found in Table 1. While exact numerical quantities vary, the studies that applied conventional farming practices found a significant proportion (≥40%) of the nitrogen applied to agricultural fields is leached to groundwater, across a range of investigated geologic settings and crops.

**Table 1:** Summary of findings from previous studies of nitrate loading under agricultural fields

Crop Type	Soil Description	Location	Measurement Method	Application rate (kg N/Ha)	Mean Estimated Loading Rate (kg N/Ha)	Percent Leached	Study Treatment Name	Reference
Corn	Sandy Loam	Michigan, USA	Drainage lysimeters	232	183	79%	CON	Martin et al. (1994)
Corn	Coarse and Fine Loamy	Michigan, USA	Soil water samplers	159	93	59%	Conventional	Syswerda et al. (2012)
Inbred Corn	Sand and Sandy Loam	Michigan, USA	Plant matter analysis and Drainage lysimeters	202	81	40%	202N	Rasse et al. (1999)
Sweet Corn	Loamy Sand	Wisconsin, USA	On‐field MLSs	250	165	66%	–	Stites and Kraft (2001)
Sweet Corn	Loamy Sand	Wisconsin, USA	N Budget	250	179	72%	–	Stites and Kraft (2001)
Sweet Corn	Loamy Sand	Wisconsin, USA	On‐field MLSs	192	148	77%	–	Kraft and Stites (2003)
Sweet Corn	Loamy Sand	Wisconsin, USA	N Budget	192	142	74%	–	Kraft and Stites (2003)
Potato	Loamy Sand	Wisconsin, USA	On‐field MLSs	327	228	70%	–	Stites and Kraft (2001)
Potato	Loamy Sand	Wisconsin, USA	N Budget	327	263	80%	–	Stites and Kraft (2001)
Raspberry	Silty over Sand and Gravelly	British Columbia, Can.	Edge‐of‐field MLSs	331	174	53%	Conventional	Kuipers et al.
Wheat	Coarse and Fine Loamy	Michigan, USA	Soil water samplers	68	61	90%	Conventional	Syswerda et al. (2012)

In this paper, we report on a field monitoring approach and analysis method that builds on the “flux fence” approach described above (Kuipers et al. 2014; Malekani et al. 2018). Our approach differs from most prior nitrogen leaching quantification approaches in the following ways. First, our permanent experimental setup is entirely employed at the edge of a field, meaning that no equipment is installed on or beneath the monitored plot. Secondly, we employ a rigorous conceptual model that analyzes both nitrate concentrations and tracer arrival times to determine the appropriate spatio‐temporal integral of nitrate concentrations and obtain nitrogen loading from an agricultural year.

The goal of this study is to provide a method for estimating the per‐field‐area average rate of nitrogen leaching, which is a regulatory threshold being proposed in Wisconsin to ensure continued availability of high‐quality drinking water in agricultural watersheds. In particular, Wisconsin's NR‐151 draft standards propose a guideline of “less than 2.2 pounds per acre per inch of groundwater recharge” which is quantitatively equivalent to 1 kg/ha/cm of recharge, and equivalent to maintaining a volume‐weighted average concentration of 10 mg/L NO_3_‐N in recharged groundwater (https://dnr.wisconsin.gov/topic/nonpoint/nr151nitrate.html). These edge‐of‐field groundwater nitrate calculations may similarly represent a “worst case” in the sense that they do not take into account possible downstream denitrification effects and assume nitrate behaves conservatively below the water table.

## Conceptual Approach

### Experiment Description

**Figure 1:**
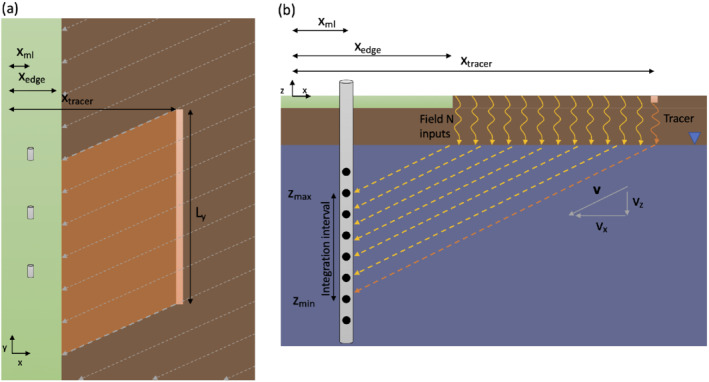
Conceptual model used for nitrate loading calculations. (a) Map view of conceptual model showing coordinate/length definitions, horizontal components of groundwater flow (dashed lines), multi‐level monitoring wells (cylinders), and tracer injection line (orange line of length *L*_y_). Brown regions represent cultivated field, while green regions represent off‐field locations. (b) Cross‐sectional view of conceptual model, showing coordinate/length and flow vector definitions, vertical groundwater flow (dashed lines), multi‐level monitoring wells (cylinder with circles representing different ports), and tracer (orange)/nitrate (yellow) flow pathways.

Especially in areas with significant pre‐existing nitrate contamination, assessing an individual area's contribution to nitrogen loading requires that the field's nitrogen contributions are separated from groundwater that did not originate on‐field, or originated in prior years. To perform this separation, our experimental approach follows the idea shown diagrammatically in Figure 1. The monitoring for this experiment takes place at a set of MLSs oriented parallel to the edge of the agricultural field. We define our coordinate system in this experiment such that the *x*‐axis and *y*‐axis are perpendicular and parallel to the field edge, respectively, and with *z* representing elevation relative to a datum (in our case, the land surface, since topography is minimal in this area). The *x* axis, and the line of installed MLS samplers, should be setup so that it is roughly perpendicular to the groundwater flow direction; as we will discuss later, however, it is not necessary that the groundwater flow direction be perfectly known. Multi‐level monitoring is performed along a line located a short distance from the edge of the field, $x=x_{\mathrm{ml}}$, in our case defined with $x_{\mathrm{ml}}=0$. At a set location $x_{\text{tracer}}$ upgradient of the multilevel monitoring system or systems, and at a time designated $t_{0}=0$, an inert tracer is applied along a line of length $L_{y}$ on the agricultural field. Nitrogen and tracer concentrations are monitored in all monitoring ports of the multilevel sampling array(s). The elevation and timing of the tracer arrival allows nitrogen concentrations to be separated into those in front of/behind $x_{\text{tracer}}$, and allows determination of horizontal and vertical transport velocities of nitrate within groundwater. Based on this information, nitrogen concentrations measured at the field edge can be associated with nitrogen fertilizer that was applied to the on‐field area (A) during the current agricultural year. Fundamentally, the conceptual model assumes that recharge to the water table arrives in a layer‐cake fashion on top of existing groundwater and thus pushes pre‐existing groundwater lower in a piston‐like fashion, which is then advected under natural groundwater gradients that are presumed to be primarily horizontal.

Measurements taken at the field edge consist of nitrate and tracer concentrations at monitoring ports over time ($C_{NO_{3}-N}\left(z,t\right)$ and $C_{\text{tracer}}\left(z,t\right)$, respectively). Based on the testing geometry and this data, the following analysis steps are used to determine nitrogen loss from the field area A. First, measured tracer concentrations are processed to select the time ($t_{\text{arrival}}$) and elevation ($z_{\text{arrival}}$) at which the peak tracer concentration arrives. Based on this information, derived values including groundwater transport velocities can be determined. The key experimental setup parameters, measurements, and derived values are summarized in Table 2.

**Table 2:** Measured and Derived Quantities Used in Calculating Nitrogen Loss Loading to Groundwater

Variable (Units)	Definition
Experimental parameters	
$x_{\text{edge}}\;\left(m\right)$	Lateral location of field edge
$x_{\text{tracer}}\;\left(m\right)$	Lateral location of tracer injection line
$x_{\mathrm{ml}}\;\left(m\right)$	Lateral *x* coordinate of multilevel samplers
$L_{y}\;\left(m\right)$	Length of tracer application line
$t_{0}\left(\text{days}\right)$	Time of tracer application. Using $t_{0}$ = 0, all time measurements represent duration from time of tracer application.
$t_{\text{vadose}}\left(\text{days}\right)$	Travel time from point of tracer application, vertically, to the water table. If tracer is applied at the water table, $t_{\text{vadose}}=0$.
Measured and derived quantities	
$z_{\text{arrival}}\;\left(m\right)$	Elevation of arrival of peak tracer concentration
$z_{\mathrm{wt}}\;\left(m\right)$	Elevation of the water table
$t_{\text{arrival}}\left(\text{days}\right)$	Time of arrival of peak tracer concentration
$v_{z}\left(m/\mathrm{day}\right)$	Vertical solute velocity in saturated zone $v_{z}=\left(z_{\text{arrival}}-z_{\mathrm{wt}}\right)/\left(t_{\text{arrival}}-\left(t_{0}+t_{\text{vadose}}\right)\right)$
$v_{x}\left(m/\mathrm{day}\right)$	Lateral (*x*‐direction) solute velocity in saturated zone $v_{x}=\left(x_{\mathrm{ml}}-x_{\text{tracer}}\right)/\left(t_{\text{arrival}}-\left(t_{0}+t_{\text{vadose}}\right)\right)$
S(−)	Slope (dip angle) of solute flow $S=v_{z}/v_{x}$
$A\left(m^{2}\right)$	Field area contributing to agricultural year nitrogen losses $A=\left(x_{\text{tracer}}-x_{\text{edge}}\right)L_{y}$

### Mathematical Calculation of Nitrogen Loss

Using the same conceptual approach as flux fences that have been applied at industrially contaminated sites, the total mass of nitrate passing through a control plane oriented perpendicular to the *x*‐axis can be calculated as:

(2) $$M=∭C_{NO_{3}-N}\left(z,t\right)q_{x}\text{dtdydz}$$

where $q_{x}\left(m/\mathrm{day}\right)$ is the specific discharge for the *x* direction, noting that we inherently assume that groundwater flow is steady throughout the measurement campaign. The bounds of integration are determined based on the question of interest. In the case of our study, we are interested in the nitrogen mass exiting the field that originated from the spatial area A during the current agricultural year. To account for nitrate flows that originated on the agricultural field between the tracer source and the field edge, only the nitrate measurements from the following monitoring elevations should be included (see Figure 1, cross section):

(3) $$z_{\max}=z_{\mathrm{wt}}-S\left(x_{\text{edge}}-x_{\mathrm{ml}}\right)$$

(4) $$z_{\min}=z_{\text{arrival}}$$

These represent the spatial bounds of integration across which solute concentrations should be summed. In terms of the temporal integral, for any given elevation z, our conceptual model implies that nitrate associated with leaching from a single agricultural year will arrive at the given monitoring port between the times:

(5) $$t_{\min}\left(z\right)=t_{0}+t_{\text{vadose}}+\frac{z-z_{\mathrm{wt}}}{v_{z}}$$

(6) $$t_{\max}\left(z\right)=t_{0}+t_{\text{vadose}}+\frac{z-z_{\mathrm{wt}}}{v_{z}}+365$$

These bounds of integration allow calculation of nitrogen loading over a single agricultural year; however, other bounds of integration could be employed if shorter or longer temporal intervals were desired. Working under the additional assumption that measured concentrations do not vary (or have already been averaged) along the *y*‐axis, the mass contributed by field area A over an agricultural year can be computed as:

(7) $$M=\int_{z_{\min}}^{z_{\max}}\int_{t_{\min}\left(z\right)}^{t_{\max}\left(z\right)}C_{{\mathrm{NO}}_{3}-N}\left(z,t\right)q_{x}\text{dtdz}\cdot L_{y}$$

Finally, rather than the net mass exiting the field, we normalize by the field area contributing to this mass discharge, using knowledge of the contributing field area:

(8) $$\frac{M}{A}=\frac{1}{\left(x_{\text{tracer}}-x_{\text{edge}}\right)}\int_{z_{\min}}^{z_{\max}}\int_{t_{\min}\left(z\right)}^{t_{\max}\left(z\right)}C_{{\mathrm{NO}}_{3}-N}\left(z,t\right)q_{x}\text{dtdz}$$

With the exception of $q_{x}$, all quantities in this computation are either directly specified by the experimental geometry, or determined as a derived quantity from tracer observations, as shown in Table 2. Obtaining a value for $q_{x}$ in practice requires that either: (1) Aquifer hydraulic conductivities and groundwater gradients have been estimated, such that $q_{x}$ can be calculated as $q_{x}=-K\frac{\mathrm{dh}}{\mathrm{dx}}$; or (2) that aquifer mobile porosity $\theta_{m}$ has been determined such that $q_{x}$ can be calculated as $q_{x}=\theta_{m}v_{x}$. Given that both average hydraulic conductivity and lateral hydraulic gradients can be difficult to constrain without significant effort, we focus on the latter strategy for estimating specific discharge.

## Field Experiment

### Site Setting and Initial Characterization

Our field study was carried out in southeastern Sauk County, Wisconsin USA with the goal of constraining nitrogen losses from an active agricultural field. Sauk County was chosen because it represents a relative hotspot for groundwater nitrate concentrations in Wisconsin. Based on data from UW‐Stevens Point, 16% of private well water samples in Sauk County showed concentrations above the 10 mg/L EPA standard, and maximum concentrations of almost 50 mg/L have been measured. In several townships of Sauk County bordering the Lower Wisconsin River, a particularly high prevalence of groundwater nitrate has been observed, with between 26% and 42% of samples exceeding EPA standards in these regions, on a township‐by‐township basis (UW‐Stevens Point, 2021).

The particular field area for this research, referred to as Field 19, is a center‐pivot irrigated agricultural field with a rotation including seed corn, potato, and snap beans. It is located in the valley of the Wisconsin River, with the valley floor gently sloping toward the river channel and little overall relief (Clayton and Attig, 1990). Field 19 has sandy and well drained soils; specifically, the majority of the field consists of Sparta loamy sand (USDA Web Soil Survey, 2018). The soils at the site are underlain by 100–250 ft of unlithified sand and gravel glacial outwash (Gotkowitz et al., 2005). Beneath the water table, sediments are generally observed to be clean sands with limited availability of organic matter or reduced iron that could contribute to denitrification. The water table at Field 19 is consistently roughly 1.8 m below ground surface (bgs), though occasionally as shallow as 1.2 m bgs, and is influenced by precipitation events as well as by changes to the stage of the nearby Wisconsin River.

Climatologically, Spring Green receives an average of roughly 90 cm of precipitation annually, with temperatures ranging from below freezing (<0°C) in winter to above 25°C in the summer. Rainfall can be highly variable, with the majority of monthly precipitation often occurring in several large (>2.5 cm) daily rain events. Weather data for the region, including precipitation and temperature information, is available from the Tri County Airport in Lone Rock, WI, accessible through the National Centers for Environmental Information (NOAA, Station ID USW0014921) database. The Tri County Airport is located less than 16 km (10 miles) from Field 19. Irrigation data for Field 19 have been provided to researchers by the cooperative farmers who operate Field 19. Irrigation information includes depth of water applied per date of application for the 2016–2020 growing years. Irrigation is applied via center pivot in response to periods without precipitation events and generally less than 1.3 cm (1/2 in.) depth of water at a time.

The work presented in this study is part of a larger field research effort at Field 19 funded by the Wisconsin Department of Natural Resources (DNR) and the EPA wellhead protection program. As such, a significant quantity of background data was collected in advance of nitrogen loss monitoring efforts to better characterize the study area. In particular, experiments were conducted: (1) to estimate saturated zone parameters including hydraulic gradients and their direction, groundwater transport velocity, and hydraulic conductivity; and (2) to characterize dynamics of nitrogen movement out of the vadose zone. We summarize these prior efforts below.

Using four “cardinal direction” (N/S/E/W) multi‐level wells surrounding the site, Krause (2017) monitored local water table dynamics and determined the hydraulic gradient to be 0.0006–0.00065 at Field 19, with a variable flow direction toward the southwest or toward the southeast of the field. The variability in flow direction is attributed to changes in the river stage of the nearby Wisconsin River, as the study site lies close to a large meander of the river. Krause (2017) also used slug tests in these wells to characterize hydraulic conductivity of the aquifer beneath Field 19, and found the hydraulic conductivity (*K*) to range from 16 to 75 m/day (52–246 ft./day), with an average *K* of 31 m/day (102.5 ft./day). Finally, early work also included a small pilot tracer test near the field edge to estimate the advective velocity of solutes under the field. For this, Krause (2017) used a saline solution and monitored the electrical conductivity of the groundwater via sampling and Electrical Resistivity Tomography (ERT). This pilot tracer test estimated an advective horizontal velocity of 0.17 m/day (0.55 ft./day).

**Figure 2:**
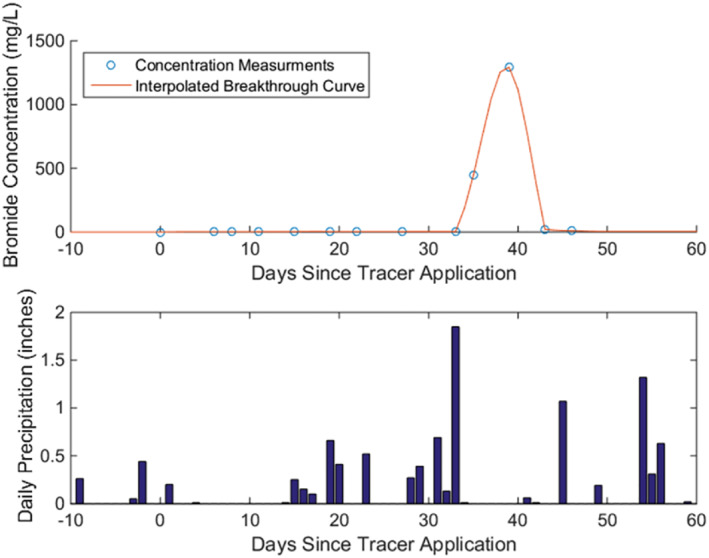
Results from an unsaturated zone tracer experiment. Top: Tracer breakthrough at the water table following application at the land surface; Bottom: precipitation data from Lone Rock Airport weather station.

In order to better understand dynamics of solute transport in the vadose zone, an unsaturated zone tracer test was also performed by Krause (2017). In this test, sodium bromide (NaBr) tracer was applied to the land surface near one of the cardinal water table monitoring wells, and the arrival of bromide was monitored regularly at the water table. Krause (2017) found the tracer to arrive at the water table 39 days after application at the land surface, following a large >3 cm precipitation event by roughly 6 days (Figure 2). Independently, laboratory column leaching experiments verified that the bromide tracer behaved similarly to nitrogen fertilizers applied at Field 19, in terms of response to simulated rainfall events.

### Nitrogen Loss Quantification Experiment

**Figure 3:**
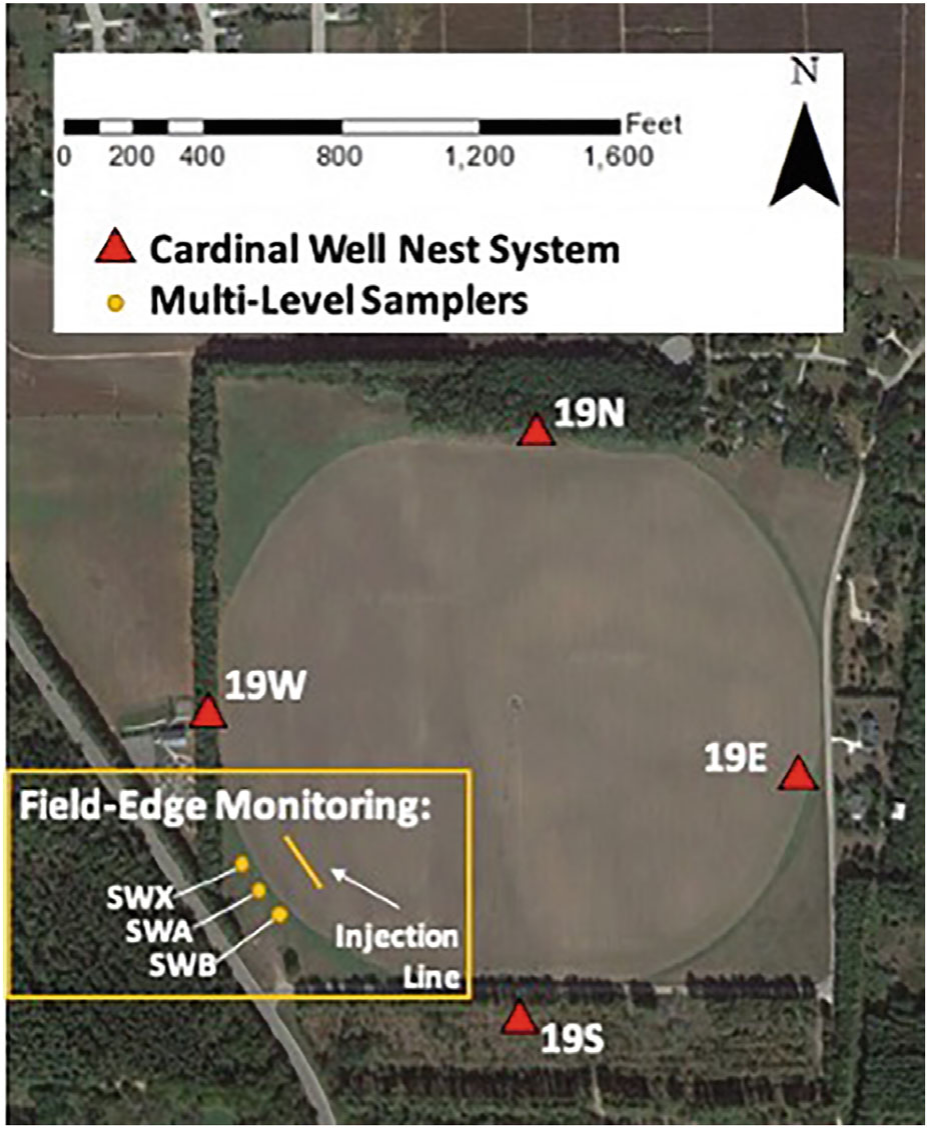
Areal view of Field 19 showing experimental well set‐up including cardinal well system and field‐edge monitoring system, with tracer injection line indicated.

The preliminary data described above was used to design an appropriately scaled implementation of our edge‐of‐field nitrogen quantification method. Based on the characterization of average groundwater flow directions from cardinal monitoring wells, it was determined that the most appropriate area for applying our methodology was at the southwest corner of Field 19. However, given the apparent flow direction variability at the site, we elected to install two sets of multi‐level monitoring systems (19‐SWA and 19‐SWB, see Figure 3) which were installed on August 15, 2016, shortly after the first tracer experiments began. The multilevel monitoring systems each consist of three closely spaced 2.54 cm (1‐in.) boreholes installed via the direct‐push method; within each borehole is a bundle of seven sampling tubes of 6 mm inner diameter (i.e., ¼ inch ID) that terminate at different elevations, each roughly 46 cm (1.5 ft) apart. Each set of multi‐level systems thus contains a total of 21 sampling intervals, with each sampling interval spaced about 46 cm apart, from near water table elevation—roughly 1.8 m (6 ft) below ground surface (bgs)—to nearly 12 m (40 ft) bgs. These intervals are numbered consecutively starting from the most shallow, e.g., 19‐SWA‐01 represents the shallowest monitoring port and 19‐SWA‐21 representing the deepest monitoring port in the 19‐SWA multilevel system. 19‐SWA and 19‐SWB are roughly 10 m (33 ft) apart, with 19‐SWB to the southeast of 19‐SWA. In order to further ensure tracer capture for future years' tracer experiments, an additional multilevel sampling system, 19‐SWX, was added the following year on November 22, 2017, roughly 10 m northwest of 19‐SWA.

Each year, tracer applications were timed to occur at or near the time of crop planting, such that a full growing year was captured. An exception to this timing was 2020, however, when spring fieldwork was impaired by the COVID‐19 pandemic. In this case tracer was applied soon after crop planting. The format of the tracer application varied from year to year, in order to test different strategies. In the 2016 growing season, tracer was applied at the water table—the “water table method” in a 30.5 m (100 ft) line perpendicular to the expected groundwater flow gradient (see Figure 3). The water table was accessed by a series of 1.8 m (6 ft) augered holes along the injection line, and tracer was injected within these holes. The dimensions of the injection line were chosen in order to maintain feasibility for application within the span of a day, while also attempting to ensure that the bromide solute plume would intersect the field‐edge monitoring array, given flow direction uncertainty. Impact to the crops was minimized by augering along a line that was already impacted by tire tracks from the center‐pivot irrigation system. The concentration of the injected bromide tracer was roughly 2400 mg/L. For the 2017 growing season, the bromide tracer was applied as a solid salt buried just below the root zone of the field—the “land surface method”. Here, 170 g of NaBr salt was placed in each of 65 shallow holes (roughly 1 m apart each, and 46 cm deep) along the same injection line as the year before. Based on limited success with the land surface method of application, the tracer was applied using the water table method for all future years. The main experimental parameters used in nitrogen loss calculations, as defined above for the conceptual model, are summarized in Table 3.

**Table 3:** Experimental Parameters for 3 Years of Bromide Injections

Growing Year	2016	2017	2018	2019	2020
$x_{\text{edge}}\;\left(m\right)$	3				
$x_{\text{tracer}}\;\left(m\right)$	57.9				
$L_{y}\;\left(m\right)$	30.5				
Injection date $t_{0}$	April 30, 2016	May 11, 2017	May 10, 2018	May 7, 2019	June 24, 2020
Tracer method	Water table	Land surface	Water table	Water table	Water table
Estimated $t_{\text{vadose}}\;\left(\text{days}\right)$	0	39	0	0	0

Other than the injection of bromide, field work consisted of routine monitoring for nitrogen and bromide in groundwater. Throughout the 5 years of experimentation, water was sampled from the multilevel system(s) via peristaltic pump approximately monthly. The methodology used to analyze nitrate concentrations in samples varied somewhat throughout the 5 years of data collection. The majority of samples were analyzed by the state‐certified Wisconsin Water and Environmental Analysis Laboratory (WEAL). In some cases, however, samples were analyzed via nitrate ion‐selective electrode or via colorimetric nitrate test strips (Hach).

Bromide sampling was carried out each year with the majority of sampling beginning at roughly 7 months after tracer injection, when bromide arrival was predicted given expected aquifer travel times. If any bromide concentrations above low background values were encountered during monthly sampling, the sampling schedule was updated to be performed more frequently, in order to ensure that the timing of the bromide peak concentration was accurately measured.

## Data

### Bromide Data

The results of bromide monitoring are summarized in Table 4. For all derived quantities, we used a water table elevation of z=−1.8m, using the land surface as our elevation datum. In general, a distinct peak of bromide arrival could be detected at a single MLS sampling port, though elevated concentrations were also often seen at surrounding ports, suggesting impacts of vertical transverse dispersion. In some cases, a later secondary peak of bromide was measured at shallower monitoring ports—these were generally lower concentrations than the initial peak and presumed to reflect bromide that had been temporarily trapped and transported near the vadose zone post‐injection, and thus were not used in any computations. Overall, the results of all 5 years of testing indicate relatively stable groundwater velocities, though the velocities determined were somewhat larger than the 0.17 m/day determined by initial site characterization. The direction of flow varied enough to cause variability in the MLS installation where peak breakthrough was measured during each experimental year, suggesting some impact from changes associated with the hydraulic gradient of the nearby Wisconsin River. Measurements of bromide during the 2017 growing season also show that application of bromide at the land surface resulted in drastically reduced concentrations, making selection of a peak arrival more uncertain. For this reason, bromide applied at the water table was used in all future years.

**Table 4:** Summary of Bromide Tracer Experiment Measurements

Growing Year	2016	2017	2018	2019	2020
Measurements					
Injection date ($t_{0}$)	April 30, 2016	May 11, 2017	May 10, 2018	May 7, 2019	June 24, 2020
Peak arrival date ($t_{\text{arrival}}$)	February 21, 2017	April 26, 2018	January 28, 2019	February 01, 2020	April 29, 2021
Peak arrival elevation ($z_{\text{arrival}}$)	−7.3 m	−8.2 m	−8.7 m	−8.8 m	−7.3 m
Total travel time	297 days	311 days^a^	264 days	270 days	309 days
Peak Br concentration measured	24.7 mg/L	5.49 mg/L	11.9 mg/L	13.5 mg/L	31.2 mg/L
Peak arrival port	19‐SWA‐14	19‐SWA‐16	19‐SWB‐16	19‐SWX‐16	19‐SWB‐13
Derived quantities					
$v_{x}\left(m/\mathrm{day}\right)$	0.19	0.19	0.22	0.21	0.19
$v_{z}\left(m/\mathrm{day}\right)$	0.019	0.021	0.026	0.025	0.018
S(−)	0.095	0.111	0.119	0.121	0.095

^a^ Calculated saturated zone travel time assumes 39 day vadose zone travel time, as measured during unsaturated zone experiments.

Some “smearing” of the bromide plume with elevation was observed in multilevel samplers. For example, the 2016 growing year tracer broke through at its maximum concentration (24.7 mg/L) in 19‐SWA‐14 on 2/21/2017. However, bromide concentrations above 8 mg/L were also observed on that same day in the shallower ports 19‐SWA‐13 and 19‐SWA‐12. We expect that this smearing is due to vertical dispersion, and assume that the center of the plume (and thus, the assigned breakthrough elevation) corresponds with the timing of the highest concentrations measured in the first bromide peak observed.

### Nitrate Data

**Figure 4:**
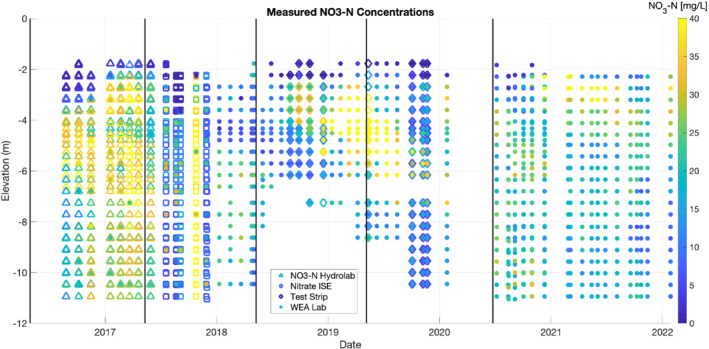
Nitrate sample results from all field monitoring. Samples from all 3 MLS installations (19‐SWA, 19‐SWB, and 19‐SWX) are represented according to monitoring point elevation. Symbols represent different methods of nitrate measurement, and black vertical lines indicate timing of annual bromide injections.

Nitrate data was generally collected monthly with a target of collecting samples from all elevations of at least one MLS system. Some gaps or reduced sampling events were associated with personnel availability, adverse weather conditions, equipment failure, and pandemic travel restrictions. The complete set of data is represented graphically in Figure 4. This dataset includes the time, elevation, and method by which nitrate concentrations were measured for all data.

The strategy for nitrate data collection varied throughout the 5 years of operation. Initially, a reliable nitrate ion‐selective electrode (ISE 1) was used to analyze most samples, with a subset of samples analyzed at the state‐certified Water and Environmental Analysis Lab (WEAL) at UW‐Stevens Point. Following failure of this ISE in summer 2017, a new ISE (ISE 2) was purchased and used throughout the remainder of 2017. Data from this ISE was found to be unreliable, and did not show good correlation with WEAL results in preliminary analyses. Thus, from 2018 forward, all collected samples were analyzed by WEAL. In some sampling from 2018 and 2019, nitrate test strips (Hach) were piloted as a confirmatory, lower‐cost approach for sample analysis. Gaps in sampling were generally associated with weather conditions—for example, the MLS sampling tubes faced difficulty with freezing at temperatures under −5°C (25 °F). A major gap in sampling was associated with university‐imposed research travel restrictions in the early phase of the COVID‐19 pandemic, during the spring of 2020.

## Results

To calculate nitrogen loading results, we used laboratory‐based (WEAL) nitrate analyses or those recorded by the reliable ion‐selective electrode (ISE 1). The integration specified in (8) was carried out numerically, as follows. We first subsampled time (t) and elevation (z) to a uniform grid of 1 day and 0.1 m, respectively, and then interpolated NO_3_‐N values to these points using linear interpolation over space and time. To approximate specific discharge $q_{x}$, we first collected precipitation (*P*) and temperature (*T*) data from the Lone Rock airport record and irrigation (*I*) data from the growers. Total (*P* + *I*) data obtained for this field ranged from 804 to 1060 mm (31.7″–41.8″). Using a Thornthwaite‐based potential evapotranspiration (PET) calculation (Thornthwaite 1948), we estimated the total monthly recharge R[m] as the remainder R=(P+I)−PET for cases where this quantity was positive (and zero otherwise). Based on these monthly estimates we calculated the recharge that took place between the time of tracer injection and tracer breakthrough. Recharge estimates obtained using our method ranged between 322 and 472 mm (12.7″–18.6″). These estimates of recharge are broadly consistent with other modeling efforts in this region. For example, Bradbury et al. (2017) performed an analysis of recharge rates in the similar Wisconsin Central Sands region using the USGS SWB modeling code. Using this alternative approach, they measured precipitation of 27.9″, 31,5″ and 36.5″ (708, 800, and 927 mm) and estimated recharge of 9.6″, 12.5″, and 16.4″ (244, 318, and 417 mm) over irrigated areas in dry, average, and wet years respectively. Our higher recharge estimates (>400 mm) represent those from 2018 and 2019, where exceptional rainfall and flooding were observed in this part of the state.

Using the bromide breakthrough elevation, we then used our recharge calculation to estimate mobile porosity as $\theta_{m}=R/\left(z_{\mathrm{wt}}-z_{\text{arrival}}\right)$. Based on the 5 years of data, annual $\theta_{m}$ estimates obtained via this method ranged from 5.0% to 7.1%. Since porosity is presumed to remain constant, we chose the average estimate across all 5 years of 6.4% to perform further computation. The remarkable consistency in effective porosity estimates (±1% porosity) across all 5 years suggests that errors and uncertainties in recharge and breakthrough elevation estimates are minimal, even though a value of 6.4% is less than might be expected for fine sand sediments. Consistent bias toward lower porosity estimates could occur in our approach if either recharge is underestimated or the elevation of bromide breakthrough is underestimated. Of these two options, we believe recharge underestimation is unlikely. Tracer breakthrough elevation bias may be introduced by initial injection of bromide being somewhat below the water table, or by density effects, causing some bias in effective porosity estimates. This possibility remains an area for exploration in future applications of our method. That said, we note that effective porosity values as low as 1% have been cited in summaries for similar sediments (Woessner and Poeter 2020), so we believe our estimate is a realistic value.

**Figure 5:**
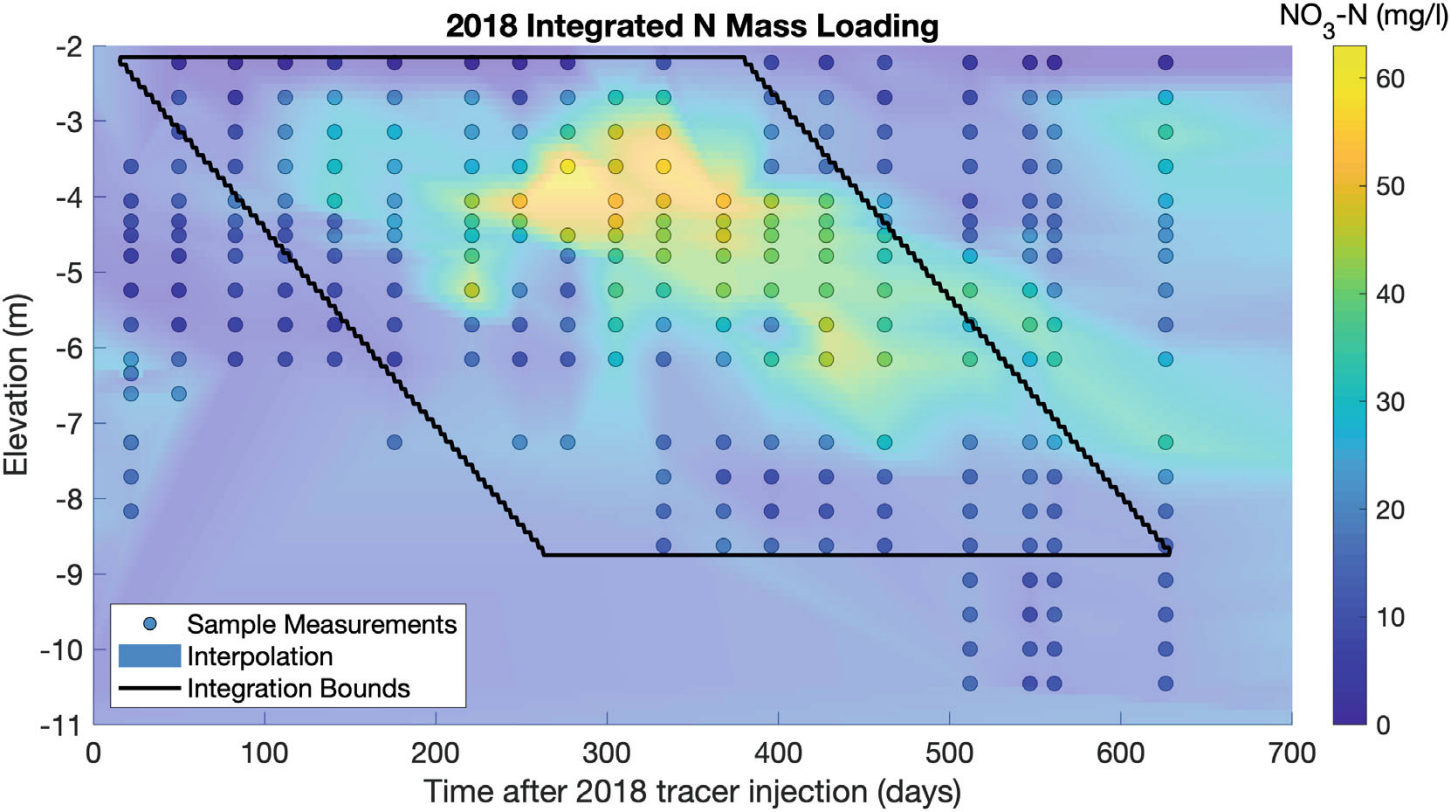
Visualization of nitrogen loading computation from integrated breakthrough for 2018 loading year. Sample measurements shown as circles, with background colors represent interpolation of concentration across elevations/time. The black parallelogram represents the spatio‐temporal bounds of integration in Equation (8), used for 2018 nitrate loading computation.

Finally, we integrated the product $v_{x}\theta_{m}C_{NO_{3}-N}$ over the appropriate bounds of integration (7) to obtain each annual nitrogen loading estimate. A graphical example of the integration performed for the 2019 growing year is shown in Figure 5. The results of nitrate loading estimation for our 5 years of data are reported in Table 5, along with farmer‐reported nutrient application rates, yields, and qualitative yield ranking. In particular, there is a qualitative negative correlation between yield and nitrogen leaching that can be observed, with high yield years seeing lower nitrogen leaching (presumably because more nitrogen is incorporated into plant biomass) and lower yield years leading to more leaching. Overall, the nitrogen leaching rates estimated for Field 19 are similar to or slightly lower than rates from similar studies on conventionally farmed corn fields (Table 1). That said, the leaching rates are high enough that the groundwater is still significantly impacted; indeed, most groundwater samples collected by our edge‐of‐field multilevel samplers were well above the 10 mg/L NO_3_‐N standard set by the EPA.

**Table 5:** Nitrogen Leaching Rates and Farmer‐Provided Nutrient Application and Crop Information

Growing Year	2016	2017	2018	2019	2020
Crop	Seed corn	Seed corn	Potato	Seed corn	Seed corn
*N* application rate (kg/ha)	222	228	302	236	230
Yield	61 bushels/acre	104 bushels/acre	282 Cwt/acre	73 bushels/acre	54 bushels/acre
Grower yield ranking	Good	High	Low	Good	Good
*N* loading estimate (kg/ha)	112	64	148	132^a^	89
Percent *N* leached to groundwater	50%	28%	49%	56%	39%

^a^ A significant number of sampling campaigns were canceled due to COVID‐19, likely reducing accuracy.

## Discussion

This work has developed a new mass flux method for estimating total nitrogen loading from a given year of agricultural field operation, with an explicit formula for calculating annually variable nitrogen loading per unit field area. Our method is expected to benefit from increased grower “buy in” given the fact that no instrumentation needs to be installed on‐field, and since disturbance to the field during the growing season is minimal. Additionally, since individual elevations measured by our field‐edge multilevel samplers (MLSs) effectively represent different lateral locations where nitrogen loading occurred (Figure 1b), we expect that our method will more accurately reflect average loading rates over a field and will be less susceptible to spatial variability issues than point‐wise measurements from, e.g., porous cups or small lysimeters. Personal experience has shown that long‐term maintenance costs for this approach are low, as well, indicating that MLS installations can be used to assess inter‐annual variability in nitrogen loading rates.

Some drawbacks of this method were also made apparent throughout the 5 years of this study, and when performing data processing. For one, gaps in the sampling campaign due to weather or other circumstances are likely to make loading estimates less accurate. Our method captures spatio‐temporal variability in nitrogen loading that is likely valuable for understanding loading dynamics, but this repeated sampling incurs significant staffing effort, labor, and analysis costs. Also, because our tracer applications at the land surface were less successful in generating clear tracer breakthrough events, we recommend that tracer be injected at the water table – this requirement restricts the range of applicability of our method to environments where the water table can be easily accessed for tracer injection. Finally, since our method (unlike large lysimeters) is inherently reliant on sample concentration measurements rather than capturing direct solute fluxes, additional assumptions or computations to estimate porosity and specific discharge are necessary. Additional uncertainty in mass loading estimates is likely associated with the empirical formulas used to estimate recharge and thus infer mobile porosity.

The nitrate loading rates found by our study (Table 5) are somewhat low compared to previous studies on corn and potato fields (Table 1), both in terms of absolute kg/ha and as percent of applied nitrogen. That said, we acknowledge that uncertainty in important quantities such as porosity estimates and NO_3_‐N concentration measurements could impact or bias this result. Our computations also find that in all years >25% of applied N is lost to groundwater, and in most years we expect that approximately half of N applied will be lost to groundwater via leaching. The operators of Field 19 already employ many modern management practices, such as split nutrient applications, cover crops, and the use of multiple in‐field management zones receiving variable rates of fertilizer. We thus consider this field as an example of a well‐managed site with all best practices adhered to. It should be noted, however, that recommended practices for nutrient application in Wisconsin focus on the economic tradeoff between fertilizer costs and increased yield (Laboski and Peters 2018), without recognizing the externally borne cost caused by environmental and health impacts of nitrogen leached to groundwater. It is unlikely that significant further reductions in nutrient loading to groundwater will be attained unless nutrient management recommendations and policies are reframed to consider the economies of environmental and public health costs.

## Conclusions and Future Work

This study demonstrated the use of edge‐of‐field multilevel monitoring systems, combined with annual tracer injections, to estimate the rate of nitrogen loss from an agricultural field. The experimental strategy employed in this study—while similar to “flux fence” methods applied at remediation sites—has not to the best of our knowledge been employed quantitatively in nutrient management studies. Use of this system required collaboration with the farmers in advance of tracer application; otherwise, the impact of this monitoring strategy on field operations was minimal. This contrasts strongly with methods that employ in‐field wells, MLSs, or lysimeters and thus require disturbance (and possibly, continuous access) to the field. This setup also minimizes the likelihood that instrumentation will be damaged by commercial farm operations throughout the several years of a study. Similar to methods employing on‐field lysimeters or wells, our approach assesses nitrogen additions in groundwater measurements, rather than performing indirect calculations of nitrogen loss which introduce greater uncertainty about actual groundwater loading. While not employed in this study, this method could be used with cooperating farmers to assess the impact of changing best management practices (BMP) on leaching to groundwater.

We consider Field 19 as an example of a well‐managed site that adheres to current recommendations and best practices. That said, the environment near Spring Green represents a challenging one for nitrogen management, given the high‐permeability sandy soils and prominence of episodic large rain events in the region. Flushing of nitrogen to the water table is difficult to avoid if fertilizer has been applied and a major storm event subsequently occurs. In conversations with the farm operators, better long‐term weather forecasts (especially to predict large rain events) were identified as having a strong potential to reduce nitrogen losses.

There are several modifications that could be made to our method if it were employed at another similar location. Injecting the tracer in a linear fashion and using two or three sets of multi‐level samplers to catch the tracer assumes a relative certainty in the direction of groundwater flow at a site. If hydraulic gradients are more uncertain, the tracer application could be modified such that it was applied in a circular or arc shape at a consistent distance from a single multi‐level monitoring well. This assumes knowledge of the direction of groundwater flow within 90 or 180° of uncertainty, which could easily be determined with local groundwater table maps for most sites. Another improvement in the method would be to reduce labor effort by employing automated samplers and possibly telemetered data collection. In longer‐term monitoring efforts, the significant up‐front capital cost of acquiring such equipment may be outweighed by the savings in field labor, and would allow more regular, higher‐frequency monitoring of nitrogen concentrations.

## Authors' Note

The authors do not have any conflicts of interest or financial disclosures to report.
